# Supplementary material for: GDF15 Promotes Cell Growth, Migration, and Invasion in Gastric Cancer by Inducing STAT3 Activation
Source: Int J Mol Sci. 2023 Feb 2;24(3):2925. doi: 10.3390/ijms24032925 (PMC9917887; doi:10.3390/ijms24032925)
Supplement: Supplementary file 1 [file ijms-24-02925-s001.zip › ijms-2143066-supplementary.pdf]

Supplementary Materials for

GDF15 Promotes Cell Growth, Migration, and Invasion in Gastric Cancer by Inducing STAT3 Activation

Mina Joo [1], Donghyun Kim [2], Myung-Won Lee [3], Hyo Jin Lee [3,4,*] and Jin-Man Kim [1,2,3,*]

[1] Department of Medical Science, College of Medicine, Chungnam National University, Daejeon 35015, Republic of Korea
[2] Department of Pathology, College of Medicine, Chungnam National University, Daejeon 35015, Republic of Korea
[3] Department of Internal Medicine, College of Medicine, Chungnam National University, Daejeon 35015, Republic of Korea
[4] Infection Control Convergence Research Center, College of Medicine, Chungnam National University, Daejeon 35015, Republic of Korea
* Correspondence: cymed@cnu.ac.kr (H.J.L.); jinmank@cnu.ac.kr (J.-M.K.); Tel.: +82-42-280-8369 (H.J.L.); +82-42-580-8237 (J.-M.K.)

Supplementary Table S1. Patient information analyzed for mRNA expression of GDF15.

Patient No.	Age (yr)	Gender	Type	H. *Pylori*
1	81	Male	Adenocarcinoma, intestinal	-
2	96	Female	Adenocarcinoma, diffuse	-
3	69	Male	Adenocarcinoma, intestinal	-
4	80	Female	Adenocarcinoma, diffuse	+

Supplementary Table S2. Baseline characteristics of the CNUH cohort.

Patient No.	GDF15 Expression	Age	Gender	Live or Death	DAYS	OS (mon)	OS (yr)	Location	Size	Differentiation	Lymphatic Invasion	Venous Invasion	T Stage
1	2	50	male	live	1912	63.73	5.3	lower	≦ 5cm	undifferentiated	invasion	invasion	1
2	2	68	female	live	2385	79.50	6.6	lower	≦ 5cm	differentiated	no invasion	no invasion	1
3	3	71	female	death	401	13.37	1.1	middle	> 5cm	undifferentiated	invasion	no invasion	4
4	2	72	male	live	1243	41.43	3.5	lower	≦ 5cm	differentiated	no invasion	invasion	1
5	2	75	female	live	1826	60.87	5.1	middle	≦ 5cm	undifferentiated	no invasion	no invasion	1
6	3	61	male	death	243	8.10	0.7	lower	≦ 5cm	differentiated	no invasion	no invasion	2
7	3	60	male	death	194	6.47	0.5	middle	≦ 5cm	differentiated	invasion	invasion	1
8	1	71	male	live	1925	64.17	5.3	middle	≦ 5cm	differentiated	no invasion	no invasion	1
9	2	64	male	live	1673	55.77	4.6	middle	≦ 5cm	undifferentiated	no invasion	no invasion	1
10	2	46	female	death	394	13.13	1.1	middle	> 5cm	undifferentiated	no invasion	no invasion	3
11	2	50	male	live	1698	56.60	4.7	middle	≦ 5cm	differentiated	no invasion	invasion	1
12	0	38	male	death	8	0.27	0.0	lower	≦ 5cm	differentiated	invasion	invasion	1
13	1	53	female	live	1951	65.03	5.4	middle	≦ 5cm	differentiated	no invasion	no invasion	1
14	1	68	male	live	1356	45.20	3.8	lower	≦ 5cm	differentiated	no invasion	no invasion	1
15	1	55	male	live	2093	69.77	5.8	middle	≦ 5cm	differentiated	no invasion	no invasion	3
16	1	45	male	live	1518	50.60	4.2	lower	≦ 5cm	undifferentiated	no invasion	no invasion	1
17	2	66	male	live	1572	52.40	4.4	middle	≦ 5cm	differentiated	invasion	no invasion	1
18	3	61	male	death	572	19.07	1.6	lower	≦ 5cm	differentiated	no invasion	no invasion	1
19	2	60	male	death	927	30.90	2.6	middle	> 5cm	undifferentiated	no invasion	no invasion	3
20	1	68	female	live	1551	51.70	4.3	lower	≦ 5cm	differentiated	no invasion	no invasion	1
21	2	43	female	live	1843	61.43	5.1	lower	≦ 5cm	differentiated	no invasion	invasion	1
22	2	69	male	death	883	29.43	2.5	lower	≦ 5cm	differentiated	no invasion	no invasion	3
23	0	62	male	death	980	32.67	2.7	middle	> 5cm	undifferentiated	no invasion	no invasion	2
24	2	75	male	death	67	2.23	0.2	lower	≦ 5cm	undifferentiated	invasion	invasion	1

25	3	54	male	death	353	11.77	1.0	lower	> 5cm	undifferentiated	no invasion	no invasion	3
26	1	63	female	death	827	27.57	2.3	lower	≦ 5cm	undifferentiated	no invasion	no invasion	3
27	2	50	male	live	1491	49.70	4.1	middle	≦ 5cm	undifferentiated	invasion	no invasion	1
28	0	68	male	live	1600	53.33	4.4	middle	≦ 5cm	undifferentiated	no invasion	no invasion	1
29	2	69	male	live	1856	61.87	5.2	lower	≦ 5cm	differentiated	no invasion	no invasion	1
30	2	74	female	death	1529	50.97	4.2	middle	≦ 5cm	undifferentiated	no invasion	no invasion	3
31	1	54	male	death	199	6.63	0.6	lower	≦ 5cm	undifferentiated	no invasion	no invasion	1
32	2	60	male	live	1240	41.33	3.4	middle	≦ 5cm	undifferentiated	no invasion	invasion	1
33	0	40	female	live	1793	59.77	5.0	middle	≦ 5cm	undifferentiated	invasion	invasion	1
34	1	66	male	live	1936	64.53	5.4	middle	≦ 5cm	differentiated	no invasion	no invasion	1
35	0	68	male	death	1434	47.80	4.0	middle	≦ 5cm	differentiated	invasion	invasion	1
36	0	60	female	live	1734	57.80	4.8	middle	≦ 5cm	undifferentiated	no invasion	no invasion	1
37	1	69	female	live	1739	57.97	4.8	lower	> 5cm	differentiated	no invasion	no invasion	2
38	2	62	male	death	873	29.10	2.4	middle	≦ 5cm	differentiated	no invasion	no invasion	1
39	3	59	male	live	1503	50.10	4.2	middle	≦ 5cm	undifferentiated	no invasion	no invasion	1
40	3	70	male	death	607	20.23	1.7	lower	≦ 5cm	differentiated	no invasion	no invasion	1
41	2	68	female	death	1168	38.93	3.2	lower	≦ 5cm	differentiated	no invasion	no invasion	1
42	0	54	male	live	2227	74.23	6.2	lower	≦ 5cm	undifferentiated	no invasion	no invasion	2
43	1	73	female	death	452	15.07	1.3	lower	> 5cm	undifferentiated	no invasion	no invasion	3
44	1	55	male	death	523	17.43	1.5	middle	> 5cm	undifferentiated	no invasion	no invasion	3
45	2	60	male	live	1541	51.37	4.3	lower	≦ 5cm	differentiated	no invasion	no invasion	1
46	2	86	male	death	46	1.53	0.1	middle	≦ 5cm	undifferentiated	no invasion	no invasion	3
47	3	39	male	death	846	28.20	2.4	middle	≦ 5cm	undifferentiated	no invasion	invasion	3
48	1	63	male	death	1167	38.90	3.2	upper	≦ 5cm	undifferentiated	no invasion	no invasion	3
49	2	63	male	death	1349	44.97	3.7	lower	> 5cm	undifferentiated	no invasion	no invasion	3
50	2	70	female	live	2443	81.43	6.8	lower	≦ 5cm	differentiated	invasion	no invasion	1
51	3	49	male	live	2025	67.50	5.6	middle	≦ 5cm	undifferentiated	no invasion	no invasion	2
52	2	58	female	live	1794	59.80	5.0	lower	> 5cm	undifferentiated	no invasion	no invasion	2
53	3	77	male	death	283	9.43	0.8	middle	≦ 5cm	undifferentiated	no invasion	invasion	3
54	3	59	male	live	2108	70.27	5.9	middle	≦ 5cm	undifferentiated	no invasion	no invasion	2
55	3	66	male	death	204	6.80	0.6	middle	≦ 5cm	differentiated	no invasion	no invasion	3
56	3	48	male	death	457	15.23	1.3	middle	> 5cm	undifferentiated	no invasion	no invasion	3
57	3	82	female	death	180	6.00	0.5	middle	≦ 5cm	undifferentiated	no invasion	no invasion	3
58	2	44	female	live	1771	59.03	4.9	lower	≦ 5cm	differentiated	invasion	no invasion	1
59	3	68	male	death	446	14.87	1.2	middle	> 5cm	undifferentiated	no invasion	no invasion	3
60	3	40	male	live	2018	67.27	5.6	middle	> 5cm	undifferentiated	no invasion	no invasion	1
61	1	43	male	live	2172	72.40	6.0	middle	≦ 5cm	undifferentiated	no invasion	no invasion	2
62	3	60	male	death	1409	46.97	3.9	upper	> 5cm	differentiated	no invasion	no invasion	3
63	1	54	male	live	1671	55.70	4.6	middle	≦ 5cm	differentiated	invasion	invasion	1
64	1	33	male	death	459	15.30	1.3	middle	≦ 5cm	undifferentiated	no invasion	no invasion	3
65	3	67	female	death	438	14.60	1.2	lower	≦ 5cm	differentiated	no invasion	no invasion	2
66	1	74	male	death	536	17.87	1.5	middle	≦ 5cm	undifferentiated	no invasion	no invasion	1
67	3	50	male	death	42	1.40	0.1	lower	> 5cm	undifferentiated	no invasion	no invasion	3
68	1	79	male	death	413	13.77	1.1	lower	≦ 5cm	undifferentiated	no invasion	no invasion	3
69	1	69	male	live	1435	47.83	4.0	lower	≦ 5cm	undifferentiated	no invasion	no invasion	1
70	0	46	female	death	653	21.77	1.8	middle	≦ 5cm	undifferentiated	no invasion	no invasion	3
71	2	70	male	death	1221	40.70	3.4	middle	> 5cm	differentiated	no invasion	no invasion	3
72	3	77	female	death	370	12.33	1.0	lower	≦ 5cm	differentiated	no invasion	no invasion	2
73	2	63	female	live	1984	66.13	5.5	lower	> 5cm	undifferentiated	no invasion	no invasion	1
74	1	50	female	death	294	9.80	0.8	upper	≦ 5cm	undifferentiated	no invasion	no invasion	3
75	1	50	female	death	1598	53.27	4.4	lower	≦ 5cm	differentiated	no invasion	no invasion	2
76	2	59	male	live	2252	75.07	6.3	lower	> 5cm	undifferentiated	no invasion	no invasion	2

77	2	63	male	death	369	12.30	1.0	middle	> 5cm	undifferentiated	no invasion	no invasion	3
78	0	67	male	death	470	15.67	1.3	middle	> 5cm	undifferentiated	no invasion	no invasion	3
79	3	66	male	death	144	4.80	0.4	lower	> 5cm	undifferentiated	no invasion	invasion	3
80	1	58	male	death	795	26.50	2.2	middle	≦ 5cm	undifferentiated	no invasion	no invasion	3
81	0	64	female	death	618	20.60	1.7	upper	> 5cm	undifferentiated	invasion	invasion	2
82	2	64	female	death	519	17.30	1.4	middle	> 5cm	undifferentiated	no invasion	no invasion	3
83	2	60	male	death	600	20.00	1.7	middle	≦ 5cm	undifferentiated	invasion	no invasion	1
84	3	62	male	death	562	18.73	1.6	upper	> 5cm	undifferentiated	no invasion	no invasion	2
85	1	63	female	death	344	11.47	1.0	lower	> 5cm	undifferentiated	no invasion	no invasion	3
86	1	55	male	live	1924	64.13	5.3	lower	≦ 5cm	undifferentiated	no invasion	no invasion	1
87	2	66	male	live	2089	69.63	5.8	middle	≦ 5cm	differentiated	no invasion	no invasion	1
88	3	54	female	live	2055	68.50	5.7	lower	≦ 5cm	differentiated	no invasion	no invasion	2
89	0	63	male	live	1828	60.93	5.1	lower	≦ 5cm	undifferentiated	invasion	invasion	1
90	1	36	male	live	1782	59.40	5.0	middle	≦ 5cm	undifferentiated	no invasion	no invasion	2
91	3	36	male	death	520	17.33	1.4	upper	> 5cm	undifferentiated	invasion	invasion	3
92	1	43	female	death	1511	50.37	4.2	lower	≦ 5cm	undifferentiated	invasion	invasion	1
93	1	52	male	live	1591	53.03	4.4	middle	≦ 5cm	undifferentiated	no invasion	no invasion	1
94	3	67	male	death	758	25.27	2.1	lower	≦ 5cm	undifferentiated	no invasion	no invasion	3
95	3	61	male	death	576	19.20	1.6	middle	≦ 5cm	undifferentiated	no invasion	no invasion	1
96	2	51	male	death	578	19.27	1.6	middle	> 5cm	undifferentiated	no invasion	no invasion	3
97	2	60	male	death	364	12.13	1.0	middle	≦ 5cm	differentiated	invasion	invasion	1
98	0	51	male	death	684	22.80	1.9	lower	≦ 5cm	differentiated	no invasion	no invasion	3
99	0	29	female	live	1888	62.93	5.3	middle	≦ 5cm	undifferentiated	invasion	invasion	1
100	2	61	male	death	748	24.93	2.1	lower	> 5cm	differentiated	no invasion	no invasion	1
101	1	58	female	live	2029	67.63	5.6	middle	≦ 5cm	differentiated	invasion	invasion	1
102	1	72	female	death	880	29.33	2.4	lower	> 5cm	undifferentiated	no invasion	no invasion	2
103	1	69	female	death	1584	52.80	4.4	lower	≦ 5cm	differentiated	invasion	invasion	1
104	3	66	male	death	762	25.40	2.1	lower	≦ 5cm	undifferentiated	no invasion	no invasion	3
105	1	53	male	live	1957	65.23	5.4	middle	≦ 5cm	differentiated	invasion	invasion	1
106	0	38	male	death	1515	50.50	4.2	middle	≦ 5cm	undifferentiated	no invasion	no invasion	2
107	2	66	male	live	1560	52.00	4.3	lower	≦ 5cm	differentiated	no invasion	no invasion	2
108	3	67	female	death	329	10.97	0.9	lower	> 5cm	undifferentiated	no invasion	no invasion	3
109	2	57	male	live	1905	63.50	5.3	lower	≦ 5cm	differentiated	invasion	invasion	1
110	2	57	female	live	1884	62.80	5.2	lower	≦ 5cm	undifferentiated	no invasion	no invasion	1
111	2	54	male	live	1832	61.07	5.1	lower	≦ 5cm	undifferentiated	invasion	invasion	1
112	3	54	male	death	1358	45.27	3.8	lower	> 5cm	differentiated	no invasion	no invasion	3
113	1	44	male	death	1518	50.60	4.2	upper	≦ 5cm	differentiated	no invasion	no invasion	1
114	2	80	male	death	1154	38.47	3.2	middle	≦ 5cm	differentiated	no invasion	no invasion	2
115	3	66	male	live	1804	60.13	5.0	lower	≦ 5cm	undifferentiated	no invasion	no invasion	3
116	3	42	female	death	531	17.70	1.5	lower	≦ 5cm	differentiated	no invasion	no invasion	1
117	2	39	male	live	1758	58.60	4.9	lower	≦ 5cm	differentiated	no invasion	no invasion	1
118	1	41	female	death	1009	33.63	2.8	middle	≦ 5cm	undifferentiated	no invasion	no invasion	3
119	3	68	male	death	107	3.57	0.3	upper	> 5cm	undifferentiated	no invasion	no invasion	3
120	2	43	male	live	1828	60.93	5.1	lower	≦ 5cm	differentiated	no invasion	no invasion	1
121	2	41	female	live	1833	61.10	5.1	middle	≦ 5cm	undifferentiated	invasion	invasion	1
122	2	55	male	death	217	7.23	0.6	middle	> 5cm	undifferentiated	no invasion	no invasion	3
123	2	61	male	live	1560	52.00	4.3	upper	≦ 5cm	differentiated	no invasion	no invasion	1
124	2	63	male	live	1764	58.80	4.9	middle	≦ 5cm	differentiated	invasion	invasion	1
125	2	52	female	live	1526	50.87	4.2	middle	≦ 5cm	differentiated	no invasion	no invasion	1
126	2	70	female	death	284	9.47	0.8	lower	≦ 5cm	differentiated	no invasion	no invasion	1
127	1	44	female	live	1622	54.07	4.5	middle	≦ 5cm	undifferentiated	no invasion	no invasion	1
128	2	59	female	live	1790	59.67	5.0	lower	≦ 5cm	undifferentiated	no invasion	no invasion	1

129	3	60	male	death	740	24.67	2.1	middle	≦ 5cm	differentiated	no invasion	no invasion	3
130	1	47	male	live	1789	59.63	5.0	lower	≦ 5cm	differentiated	invasion	invasion	1
131	3	41	male	live	1586	52.87	4.4	middle	≦ 5cm	differentiated	invasion	invasion	1
132	1	54	male	live	1751	58.37	4.9	lower	≦ 5cm	differentiated	invasion	invasion	1
133	2	58	male	death	342	11.40	1.0	middle	≦ 5cm	differentiated	no invasion	no invasion	1
134	1	72	male	death	682	22.73	1.9	middle	≦ 5cm	differentiated	invasion	invasion	1
135	0	57	female	live	1737	57.90	4.8	lower	≦ 5cm	differentiated	no invasion	no invasion	1
136	0	37	female	live	1587	52.90	4.4	middle	≦ 5cm	undifferentiated	no invasion	no invasion	1
137	1	59	male	death	458	15.27	1.3	middle	≦ 5cm	differentiated	no invasion	no invasion	1
138	2	66	male	death	7	0.23	0.0	middle	≦ 5cm	differentiated	no invasion	no invasion	1
139	0	49	male	live	1285	42.83	3.6	lower	≦ 5cm	differentiated	invasion	invasion	1
140	1	47	male	live	1714	57.13	4.8	lower	≦ 5cm	differentiated	invasion	invasion	1
141	2	69	female	death	1107	36.90	3.1	middle	≦ 5cm	differentiated	invasion	invasion	1
142	2	65	male	live	1662	55.40	4.6	lower	≦ 5cm	differentiated	no invasion	no invasion	1
143	3	66	male	death	1137	37.90	3.2	middle	≦ 5cm	undifferentiated	no invasion	no invasion	1
144	3	53	male	live	1671	55.70	4.6	lower	≦ 5cm	undifferentiated	no invasion	no invasion	1
145	1	69	male	death	7	0.23	0.0	middle	≦ 5cm	differentiated	invasion	invasion	1
146	2	59	male	death	168	5.60	0.5	middle	≦ 5cm	differentiated	no invasion	no invasion	1
147	0	41	male	live	1380	46.00	3.8	lower	≦ 5cm	undifferentiated	no invasion	no invasion	1
148	0	49	male	death	586	19.53	1.6	middle	≦ 5cm	differentiated	invasion	invasion	1
149	3	47	male	live	1584	52.80	4.4	middle	≦ 5cm	differentiated	invasion	invasion	1
150	3	67	male	live	1625	54.17	4.5	middle	≦ 5cm	undifferentiated	no invasion	no invasion	1
151	1	58	female	live	1369	45.63	3.8	middle	≦ 5cm	undifferentiated	no invasion	no invasion	1
152	2	39	male	death	1062	35.40	3.0	lower	≦ 5cm	undifferentiated	no invasion	no invasion	1
153	1	42	male	live	1419	47.30	3.9	middle	≦ 5cm	differentiated	no invasion	no invasion	1
154	1	69	male	live	1474	49.13	4.1	lower	≦ 5cm	differentiated	invasion	invasion	1
155	0	60	male	live	1211	40.37	3.4	lower	≦ 5cm	differentiated	no invasion	no invasion	1
156	1	72	male	death	566	18.87	1.6	middle	≦ 5cm	differentiated	no invasion	no invasion	1
157	1	59	male	death	875	29.17	2.4	middle	≦ 5cm	undifferentiated	no invasion	no invasion	1
158	1	64	male	death	326	10.87	0.9	lower	≦ 5cm	undifferentiated	no invasion	no invasion	1
159	0	45	male	death	1030	34.33	2.9	lower	≦ 5cm	differentiated	invasion	invasion	1
160	3	62	female	death	861	28.70	2.4	lower	≦ 5cm	differentiated	no invasion	no invasion	1
161	2	66	male	live	1404	46.80	3.9	lower	≦ 5cm	differentiated	no invasion	no invasion	1
162	3	50	male	live	1218	40.60	3.4	middle	≦ 5cm	differentiated	no invasion	no invasion	1
163	3	76	female	live	1504	50.13	4.2	middle	≦ 5cm	undifferentiated	no invasion	no invasion	1
164	2	59	female	live	1213	40.43	3.4	middle	≦ 5cm	undifferentiated	no invasion	no invasion	1
165	3	69	male	live	1430	47.67	4.0	lower	≦ 5cm	undifferentiated	no invasion	no invasion	1
166	2	58	male	live	1280	42.67	3.6	middle	≦ 5cm	undifferentiated	no invasion	no invasion	1
167	2	63	male	death	1003	33.43	2.8	lower	≦ 5cm	differentiated	no invasion	no invasion	1
168	3	47	female	live	1097	36.57	3.0	upper	≦ 5cm	undifferentiated	no invasion	no invasion	1
169	0	78	female	live	1344	44.80	3.7	upper	≦ 5cm	differentiated	no invasion	no invasion	1
170	3	45	male	live	1052	35.07	2.9	middle	≦ 5cm	undifferentiated	invasion	no invasion	1
171	2	40	male	death	1033	34.43	2.9	middle	≦ 5cm	differentiated	invasion	invasion	1
172	1	68	male	death	473	15.77	1.3	lower	≦ 5cm	differentiated	invasion	invasion	1
173	2	63	male	death	453	15.10	1.3	middle	≦ 5cm	differentiated	invasion	invasion	1
174	3	66	male	death	712	23.73	2.0	middle	≦ 5cm	undifferentiated	no invasion	no invasion	1
175	3	56	male	live	1330	44.33	3.7	middle	≦ 5cm	differentiated	no invasion	no invasion	1
176	2	60	male	live	1161	38.70	3.2	lower	≦ 5cm	differentiated	no invasion	no invasion	1
177	1	67	male	live	1293	43.10	3.6	middle	≦ 5cm	differentiated	no invasion	no invasion	1
178	1	69	male	death	564	18.80	1.6	middle	≦ 5cm	undifferentiated	no invasion	no invasion	1

* OS: Overall Survival.

Supplementary Table S3. Primer sequences for RT-qPCR.

Gene	Forward (5′-3′)	Reverse (3′-5′)
GDF15	CTCCAGATTCCGAGAGTTGC	ACCTGCACCTGCGTATCTCT
GAPDH	TTGATTTTGGAGGGATCTCG	GAGTCAACGGATTTGGTCGT

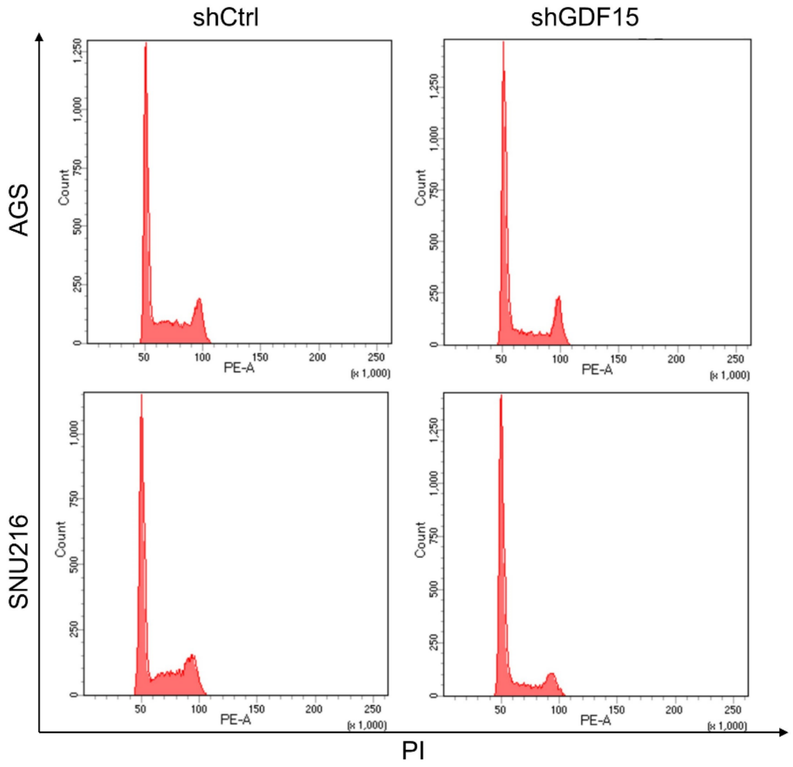

Supplementary Figure S1. Cell cycle analysis by FACS of GDF15-knockdown cells after propidium iodide (PI) staining. Knockdown of GDF15 significantly increased the proportion of cells in the G0/G1 phase com-pared with the control, whereas it decreased the proportion of cells in S phase and G2/M phase.

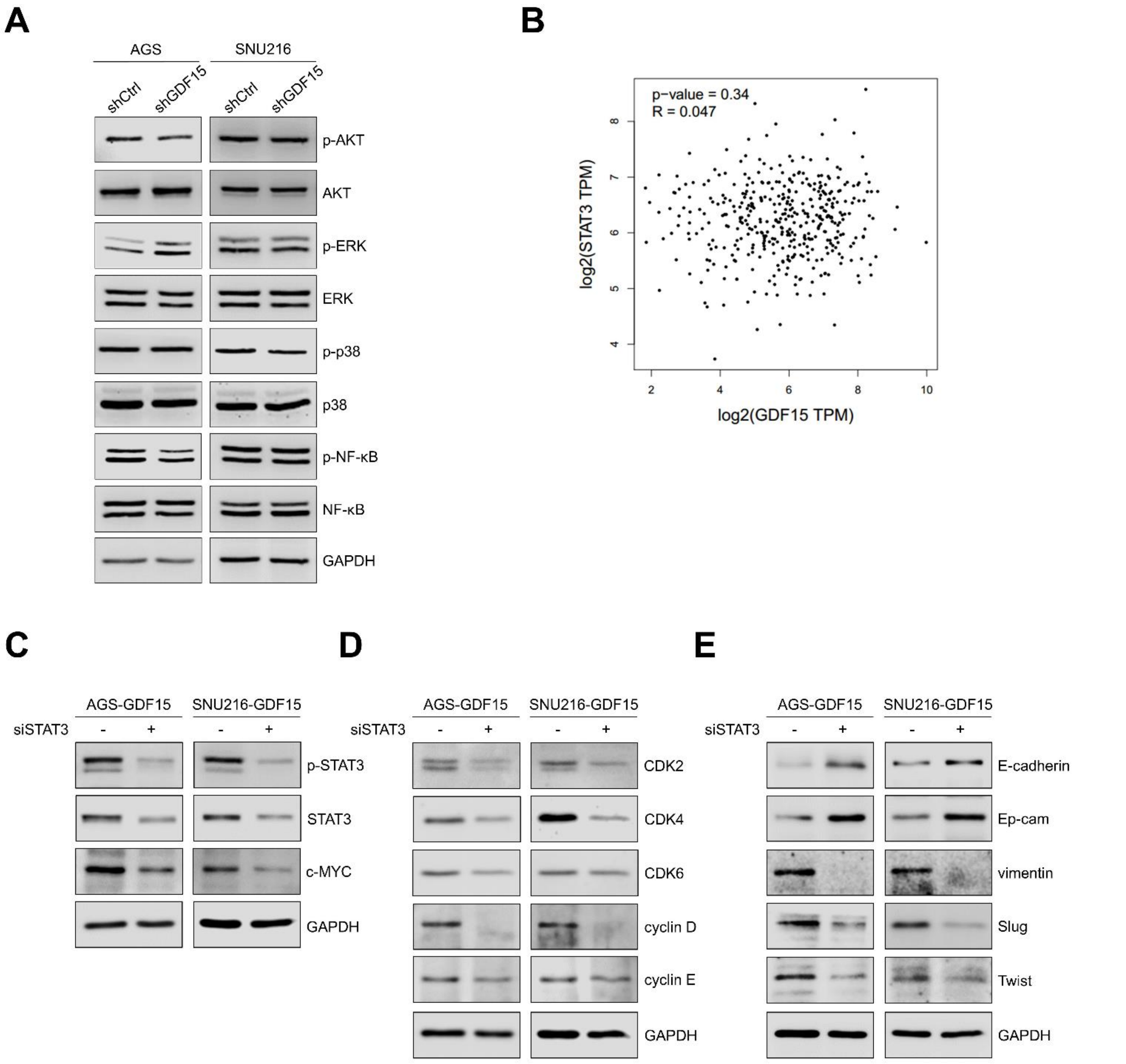

Supplementary Figure S2. (**A**) Western blotting analysis of components of several cancer-related pathways. (**B**) The correlation of GDF15 and STAT3 expression in GEPIA dataset. (**C**) Transiently transfected with control and STAT3 siRNA in GDF15-overexpressing cells. Western blotting analysis showing decreased phosphorylation of STAT3 and c-MYC. (**D**) Knockdown of STAT3 significantly downregulated cell cycle markers in GDF15-overexpressing cells. (**E**) Western blot analysis showed that STAT3 knockdown in GDF15 overexpressing cells increased expression of epithelial markers and decreased expression of mesenchymal markers.

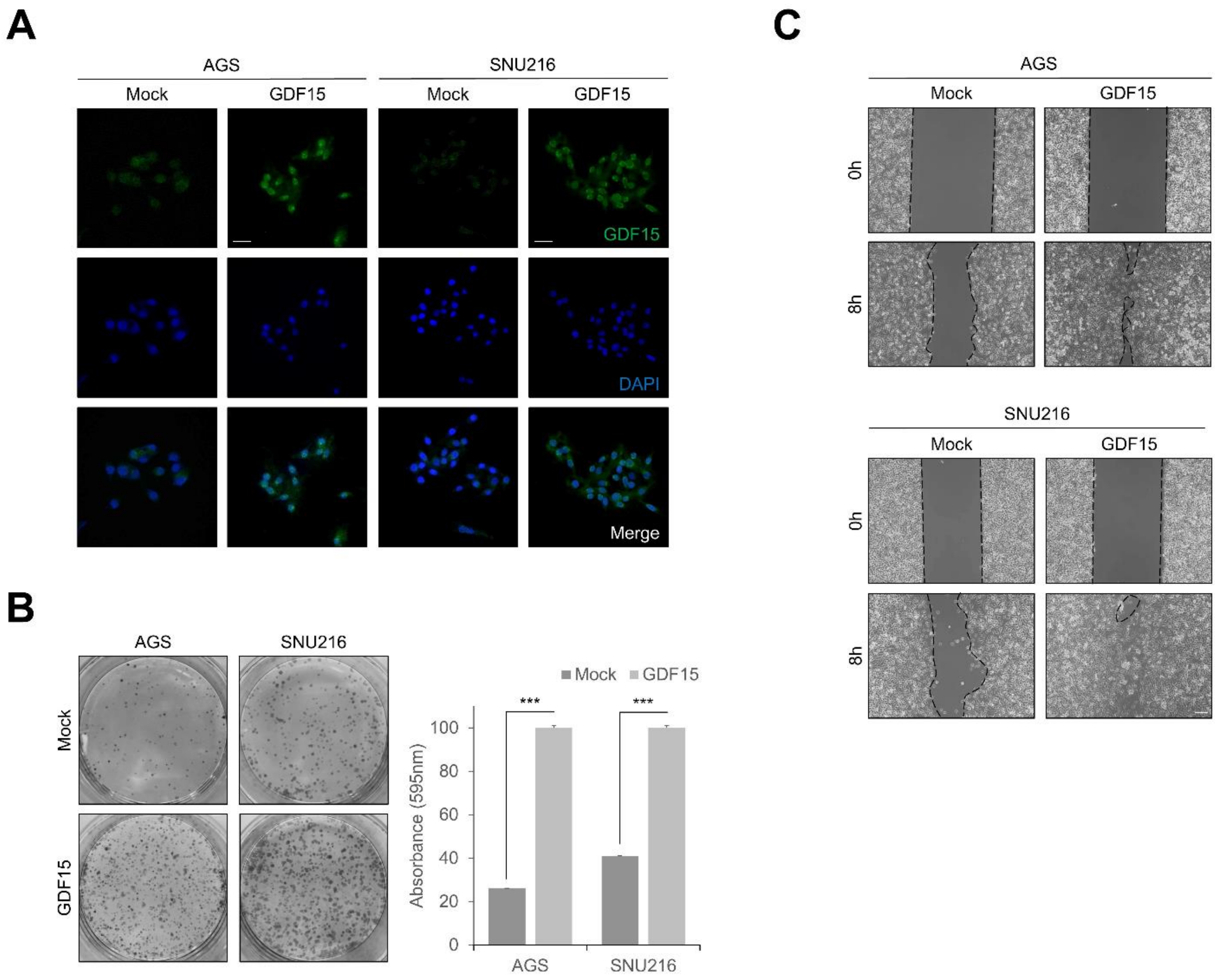

Supplementary Figure S3. (**A**) Immunofluorescence staining of GDF15 in GDF15-overexpressing cells. Scale bar, 100 μm. (**B**) The colonogenic assay was performed on a 6-well culture plate in GDF15-overexpressing cells. Crystal-violet-stained cells were dissolved in 70% alcohol, and absorbance at 595 nm was measured using a spectrophotometer. Data are presented as the mean ± SD and were evaluated using Student's t-test (n = 3). (**C**) Gap closure assay in GDF15-overexpressing cells at 0h and 8h. (n = 3) Scale bar, 100 μm. Three independent experiments were carried out in triplicate. *** $p < 0.001$.
